# Electrophysiological Effects of the Transient Receptor Potential Melastatin 4 Channel Inhibitor (4-Chloro-2-(2-chlorophenoxy)acetamido) Benzoic Acid (CBA) in Canine Left Ventricular Cardiomyocytes

**DOI:** 10.3390/ijms22179499

**Published:** 2021-08-31

**Authors:** Csaba Dienes, Tamás Hézső, Dénes Zsolt Kiss, Dóra Baranyai, Zsigmond Máté Kovács, László Szabó, János Magyar, Tamás Bányász, Péter P. Nánási, Balázs Horváth, Mónika Gönczi, Norbert Szentandrássy

**Affiliations:** 1 Department of Physiology, Faculty of Medicine, University of Debrecen, 4032 Debrecen, Hungary; dienes.csaba@med.unideb.hu (C.D.); hezso.tamas@med.unideb.hu (T.H.); kiss.denes@med.unideb.hu (D.Z.K.); baranyai.dora@med.unideb.hu (D.B.); kovacs.zsigmond@med.unideb.hu (Z.M.K.); laszlo.szabo@med.unideb.hu (L.S.); magyar.janos@med.unideb.hu (J.M.); banyasz.tamas@med.unideb.hu (T.B.); nanasi.peter@med.unideb.hu (P.P.N.); horvath.balazs@med.unideb.hu (B.H.); gonczi.monika@med.unideb.hu (M.G.); 2 Doctoral School of Molecular Medicine, University of Debrecen, 4032 Debrecen, Hungary; 3 Division of Sport Physiology, Department of Physiology, Faculty of Medicine, University of Debrecen, 4032 Debrecen, Hungary; 4 Department of Dental Physiology and Pharmacology, Faculty of Dentistry, University of Debrecen, 4032 Debrecen, Hungary; 5 Faculty of Pharmacy, University of Debrecen, 4032 Debrecen, Hungary; 6 Department of Basic Medical Sciences, Faculty of Dentistry, University of Debrecen, 4032 Debrecen, Hungary

**Keywords:** CBA, TRPM4, cardiac ionic currents, cardiac action potentials, canine myocytes, action potential voltage clamp

## Abstract

Transient receptor potential melastatin 4 (TRPM4) plays an important role in many tissues, including pacemaker and conductive tissues of the heart, but much less is known about its electrophysiological role in ventricular myocytes. Our earlier results showed the lack of selectivity of 9-phenanthrol, so CBA ((4-chloro-2-(2-chlorophenoxy)acetamido) benzoic acid) was chosen as a new, potentially selective inhibitor. Goal: Our aim was to elucidate the effect and selectivity of CBA in canine left ventricular cardiomyocytes and to study the expression of TRPM4 in the canine heart. Experiments were carried out in enzymatically isolated canine left ventricular cardiomyocytes. Ionic currents were recorded with an action potential (AP) voltage-clamp technique in whole-cell configuration at 37 °C. An amount of 10 mM BAPTA was used in the pipette solution to exclude the potential activation of TRPM4 channels. AP was recorded with conventional sharp microelectrodes. CBA was used in 10 µM concentrations. Expression of TRPM4 protein in the heart was studied by Western blot. TRPM4 protein was expressed in the wall of all four chambers of the canine heart as well as in samples prepared from isolated left ventricular cells. CBA induced an approximately 9% reduction in AP duration measured at 75% and 90% of repolarization and decreased the short-term variability of APD_90_. Moreover, AP amplitude was increased and the maximal rates of phase 0 and 1 were reduced by the drug. In AP clamp measurements, CBA-sensitive current contained a short, early outward and mainly a long, inward current. Transient outward potassium current (I_to_) and late sodium current (I_Na,L_) were reduced by approximately 20% and 47%, respectively, in the presence of CBA, while L-type calcium and inward rectifier potassium currents were not affected. These effects of CBA were largely reversible upon washout. Based on our results, the CBA induced reduction of phase-1 slope and the slight increase of AP amplitude could have been due to the inhibition of I_to_. The tendency for AP shortening can be explained by the inhibition of inward currents seen in AP-clamp recordings during the plateau phase. This inward current reduced by CBA is possibly I_Na,L_, therefore, CBA is not entirely selective for TRPM4 channels. As a consequence, similarly to 9-phenanthrol, it cannot be used to test the contribution of TRPM4 channels to cardiac electrophysiology in ventricular cells, or at least caution must be applied.

## 1. Introduction

Ion channels of the mammalian transient receptor potential (TRP) superfamily are responsible for non-specific cationic currents and many of them are present in mammalian cardiomyocytes [1]. They are subdivided into six subfamilies in mammalian cells [2]. Two members in the melastatin family, TRPM4 and 5, are unique, as those are not permeable to divalent cations like Ca^2+^ [3,4]. The TRPM4-mediated non-specific monovalent cationic current requires Ca^2+^ for its activation [4,5] with an approximately 0.5–1 µM Ca^2+^ for half effective activator concentration (EC_50_) [5,6]. Ca^2+^ sensitivity of the current is modified by phosphatidylinositol-4,5-bisphosphate (PIP_2_) [6] and calmodulin [7].

TRPM4 channels are present in cardiac tissues, including the conduction system, and are involved in several cardiac conduction disorders [8,9,10,11]. The expression of TRPM4 is weaker in atrial muscle [12] but it is also expressed to a small extent in ventricular cells [9]. Recent studies reported definitive TRPM4 expression in ventricular muscle and cardiomyocytes [13,14]. TRPM4 current contributes to pacemaking in several species including rat, mouse, and rabbit by preventing bradycardia [15], prolongs action potential duration (APD) in atrial muscle [16], and is involved in the positive inotropic effect of β-adrenergic stimulation [17]. Moreover, further highlighting the importance of TRPM4 in the ventricle, it was shown that the presence of TRPM4 reduces the angiotensin-II-induced cardiac hypertrophy [18]. On the contrary, inhibition of TRPM4 protected rat hearts from hypoxia-reoxygenation-mediated injury [13]. Since the TRPM4 channel does not differentiate between Na^+^ and K^+^, it conducts a non-specific inward current. This Ca^2+^-dependent inward current has been proposed to increase arrhythmia propensity as one of the candidates of transient inward current (I_ti_) [12]. This current can be responsible for the generation of delayed afterdepolarizations (DADs) in cells with Ca^2+^ overload, similarly to Ca^2+^-activated Cl^−^ current (I_Cl(Ca)_) [19] and Na^+^/Ca^2+^ exchange current (I_NCX_) [20]. Cardiac arrhythmias are indeed associated with TRPM4, as early afterdepolarization (EAD) was dose-dependently reduced by TRPM4 inhibition in mice [21]. Others also reported that the inhibition of TRPM4 current is potentially antiarrhythmic [12,16,22]. Recent data indicates that TRPM4 is an important player in the beneficial cardiac remodeling induced by endurance training [23]. TRPM4 has an impact in heart development as well [24]. The presence and potential function of TRPM4 in the canine heart (which is a good model for human heart cardiac electrophysiology [25,26,27]) is completely unknown.

So far, physiological and pathophysiological functions of TRPM4 were either studied in knock-out animal models [17,18] or with pharmacological approach using inhibitors (or sometimes activators) of the channels. The former method is convenient for rats and mice but practically impossible to accomplish in large mammals like dogs, a species greatly resembling human myocardium regarding its electrical properties [27,28]. The pharmacological approach, however, requires selective drug(s). In previous studies, many compounds were used for the inhibition of TRPM4 including flufenamic acid [22,29,30], glibenclamide [22,30], spermine [31], or 9-phenanthrol [16,21,32]. Unfortunately, none of these drugs were selective enough, even 9-phenanthrol possessing 20 µM as a half inhibitory concentration for TRPM4 [33], was shown to affect voltage-gated Ca^2+^ and K^+^ channels in primary cardiomyocytes in higher doses (100 µM) [21]. Moreover, we have shown that in native canine cardiomyocytes, several K^+^ currents were also blocked by 9-phenanthrol in doses of 3–30 µM [34].

Recently, 4-chloro-2-[[2-(2-chlorophenoxy)acetyl]amino]benzoic acid (CBA), a potent drug with good selectivity, was developed [35]. CBA is commercially available, and its half inhibitory concentration is 20 times lower compared to 9-phenanthrol; moreover, it reduces endogenous TRPM4 currents too. Additionally, even at 10 µM where at least 90% TRPM4 current inhibition is detected, CBA hardly influences other important channels like hERG or L-type calcium (judged by the reduction in specific antagonist binding only) [35]. As information on the specificity of CBA is very limited, the present work was designed to study the effects of CBA on action potential morphology and the underlying major ionic currents in canine ventricular cardiomyocytes. We found that TRPM4 channel is expressed in the wall of all four chambers of the canine heart, as well as in isolated left ventricular cardiomyocytes. The TRPM4 inhibitor CBA inhibited transient outward K^+^ current (I_to_) and late Na^+^ current (I_Na,L_) but not L-type calcium and inward rectifier potassium currents of the canine ventricular cells. Therefore, CBA is not entirely selective and can only be used with caution to study the role of TRPM4 in native cardiomyocytes.

## 2. Results

### 2.1. Expression of TRPM4 Protein

**Figure 1:**
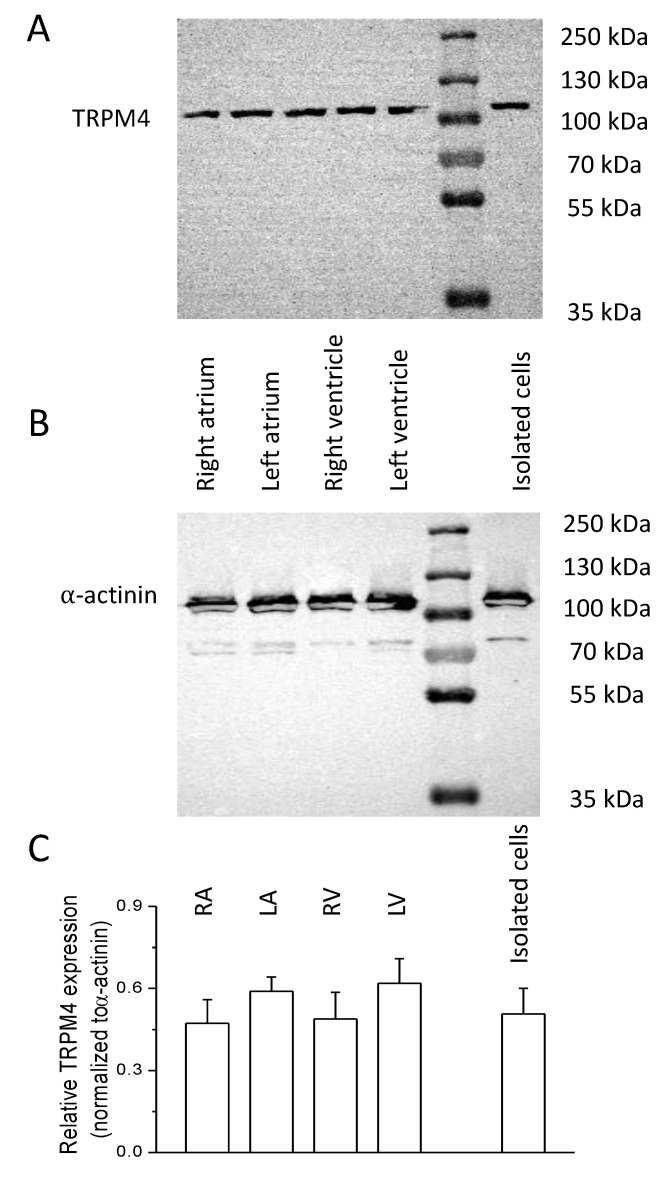
Cardiac expression of TRPM4 in thecanine heart. A and B: Representative Western blot shows TRPM4 (**A**) and α-actinin (**B**) expression in the samples indicated on the picture. The molecular weights on the right refer to the protein ladder visible in lane 5 from the left. (**C**) Average ± SEM values of relative TRPM4 expression in samples originating from the different cardiac area as indicated on top of each column. The number of animals were five in each case and at least eight independent blots were analyzed.

For protein expression studies, tissue samples and left ventricular isolated cells were obtained from five animals. Protein separation with electrophoresis and the following labeling with appropriate antibodies were made at least twice for each sample. TRPM4 could be detected in all 4 chambers of the heart as well as in isolated left ventricular cells (Figure 1). TRPM4 expression was normalized to that of α-actinin in each case. The relative expression was 0.47 ± 0.08, 0.59 ± 0.05, 0.49 ± 0.10, 0.62 ± 0.09, and 0.51 ± 0.09 in the case of tissues from the right atrium, left atrium, right ventricle, left ventricle, and isolated left ventricular cells, respectively. A significant difference could not be obtained between any of the previous samples.

### 2.2. Effects of CBA on Action Potential Morphology

**Figure 2:**
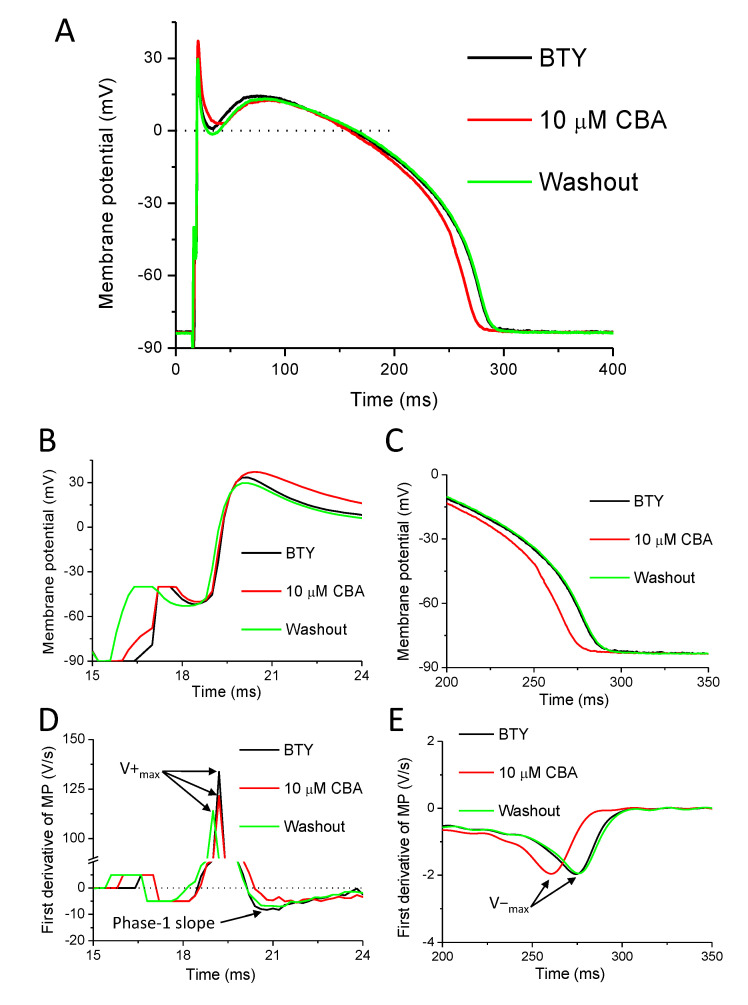
Effect of CBA on action potentials (APs) of canine left ventricular cells. (**A**) Representative APs recorded in control conditions (black), in the presence of the TRPM4 inhibitor (4-chloro-2-(2-chlorophenoxy)acetamido)benzoic acid (CBA), (10 µM) (red) and after washout of CBA (green). (**B**) The initial part of the APs is shown enlarged. (**C**) The terminal repolarization of the APs is shown enlarged. (**D**) The first derivative of curves on panel B illustrating the maximal rates of depolarization (V^+^_max_) and early repolarization (phase-1 slope). (**E**) The first derivative of curves on panel C illustrating the maximal rate of late repolarization (V^−^_max_). Stimulus artefacts and corresponding signal parts on panels (**B**,**D**) were removed for better visibility and the curves of panel (**E**) were smoothed.

**Figure 3:**
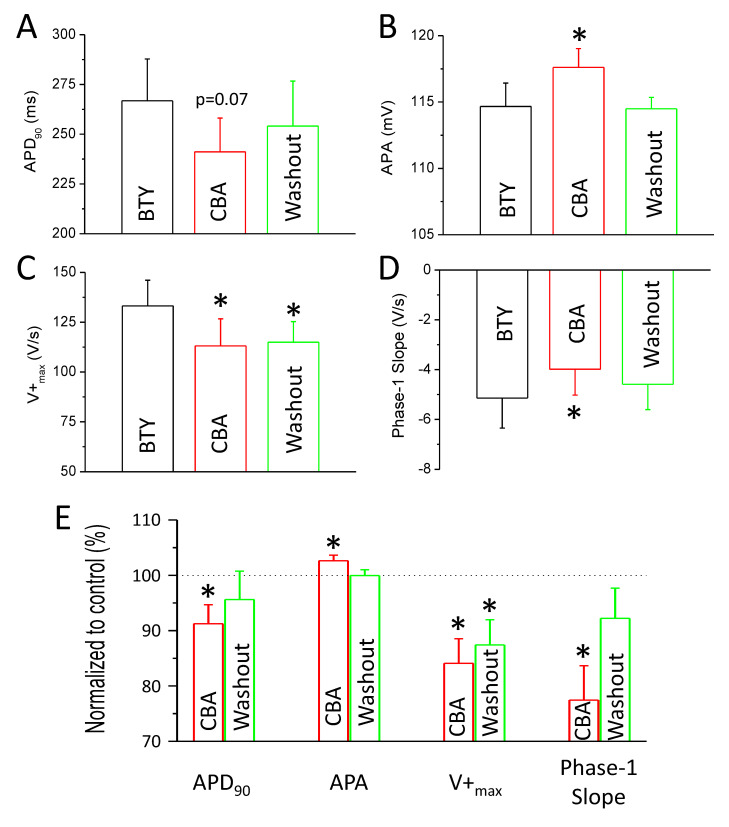
Action potential parameters affected by CBA. Diagrams showing AP parameters in control conditions (black), in the presence of CBA (10 µM, red), and after washout of CBA (green). (**A**) Average ± SEM values of action potential duration measured at 90% of repolarization (APD_90_). (**B**) Average ± SEM values of action potential amplitude (APA). (**C**) Average ± SEM values of maximal rate of depolarization (V^+^_max_). (**D**) Average ± SEM values of maximal rate of early repolarization (phase-1 slope). (**E**) Average ± SEM values of the same four parameters normalized to control conditions in the presence (red columns) and after washout of CBA (green columns). All parameters were recorded in eight cells obtained from six animals. Asterisks show statistically significant differences compared to BTY.

**Figure 4:**
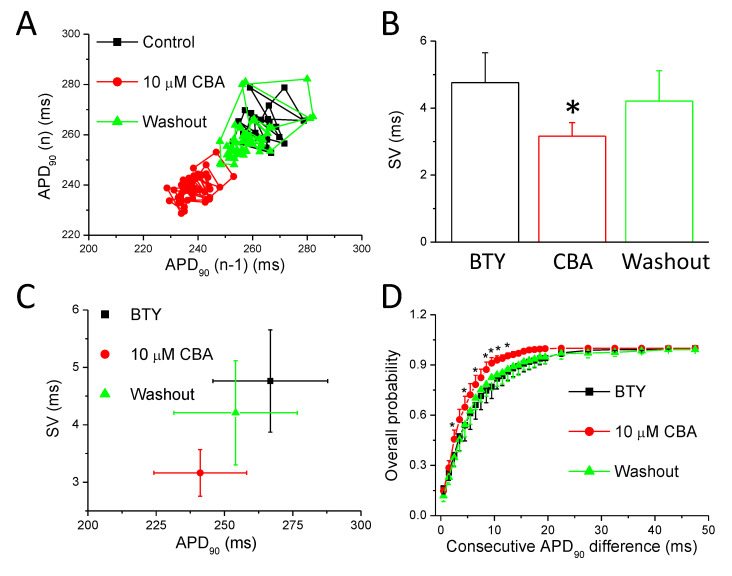
Short-term variability of ventricular repolarization. (**A**) Poincaré diagram of a representative experiment showing consecutive APD_90_ values in control conditions (black squares), in the presence of CBA (10 µM, red circles) and after washout of CBA (green triangles). (**B**) Average ± SEM values of short-term variability of ventricular repolarization (SV) obtained in eight cells isolated from six animals. (**C**) Average ± SEM values of SV as a function of average ± SEM values of APD_90_ values in control conditions (black), in the presence of CBA (10 µM, red), and after washout of CBA (green). (**D**) Overall probability of consecutive APD_90_ differences generated from eight measurements from six animals in control (black), CBA (red), and after washout (green) conditions. Asterisks show statistically significant differences compared to BTY.

APs were recorded under conditions of normal Ca^2+^ cycling using KCl-filled conventional microelectrodes believed to be the closest to the physiological situation. Eight cells isolated from six animals were exposed to 10 µM CBA for 5 min and a 5-min-long period of washout was applied at the end of the experiment. The results are presented in Figure 2, Figure 3 and Figure 4. A representative recording with AP and its first-time derivative is shown in Figure 2. CBA exerted no effect on resting membrane potential (RMP), overshoot potential (OSP), membrane potential at the half duration of APD_90_ (Plateau_50_), and the maximal rate of phase 3 (V^−^_max_) (Table 1).

There was a tendency for AP shortening in the presence of CBA at all studied repolarization levels with a duration of the AP from the peak to 50%, 75%, and 90% of repolarization, APD_50_, APD_75_, and APD_90_, respectively. APD_50_: 214.7 ± 22.3 versus 191.9 ± 18.2 ms (*p* = 0.09); APD_75_: 254.2 ± 21.2 versus 229.0 ± 17.4 ms (*p* = 0.07); APD_90_: 266.8 ± 21.0 versus 241.1 ± 17.0 ms (*p* = 0.07) in the absence and presence of CBA, respectively. CBA significantly reduced the maximal rates of depolarization (V^+^_max_: 133.2 ± 12.9 versus 113.1 ± 13.6 V/s in the absence and presence of CBA, respectively). The maximal rate of early repolarization (phase-1 slope: −5.1 ± 1.2 versus −3.9 ± 1.0 V/s in the absence and presence of CBA, respectively) of the APs of canine left ventricular cardiomyocytes was also significantly reduced; however, the value of APA was increased (114.7 ± 1.8 versus 117.6 ± 1.4 mV in the absence and presence of CBA, respectively) (Figure 3A–D). These changes were small but statistically significant for APA, phase-1 slope, and V^+^_max_. In case of APD_75_ and APD_90_, statistical significance was only reached after normalizing the absolute values to their respective controls. APD_75_ was reduced to 91.0 ± 3.6% and APD_90_ was reduced to 91.3 ± 3.4% of their respective control values in the presence of CBA (Figure 3E). Upon washout, almost all above-mentioned actions of CBA were reversible (Figure 3), the only exception being the maximal rate of depolarization as V^+^_max_ remained 114.9 ± 10.4 V/s after washout.

### 2.3. Effects of CBA on Short-Term Variability of Repolarization

Analysis of short-term variability of ventricular repolarization was also determined in the same eight studied cells. APD_90_ was used to approximate the duration of cardiac APs; the values of SV and relative SV were determined according to that described in methods. A representative Poincaré diagram in Figure 4A shows the reversible shortening of APs in the presence of 10 µM CBA. The value of SV (Figure 4B) was significantly smaller, about 27% lower in CBA compared to that of the control condition (4.8 ± 0.9 ms in control versus 3.2 ± 0.4 ms in CBA). This decrease was mostly reversible (SV after washout: 4.2 ± 0.9 ms). As was mentioned earlier, the value of SV depends on APD itself; therefore, we plotted SV values as a function of respective APD values to illustrate the action of CBA and its reversibility (Figure 4C). Moreover, the cumulative distribution curve illustrating the dispersion of differences in consecutive APD_90_ values was shifted toward smaller beat-to-beat variability in the presence of CBA in a reversible manner (Figure 4D).

### 2.4. Effects of CBA Measured with a Canonic Ventricular AP Using the APVC Technique

**Figure 5:**
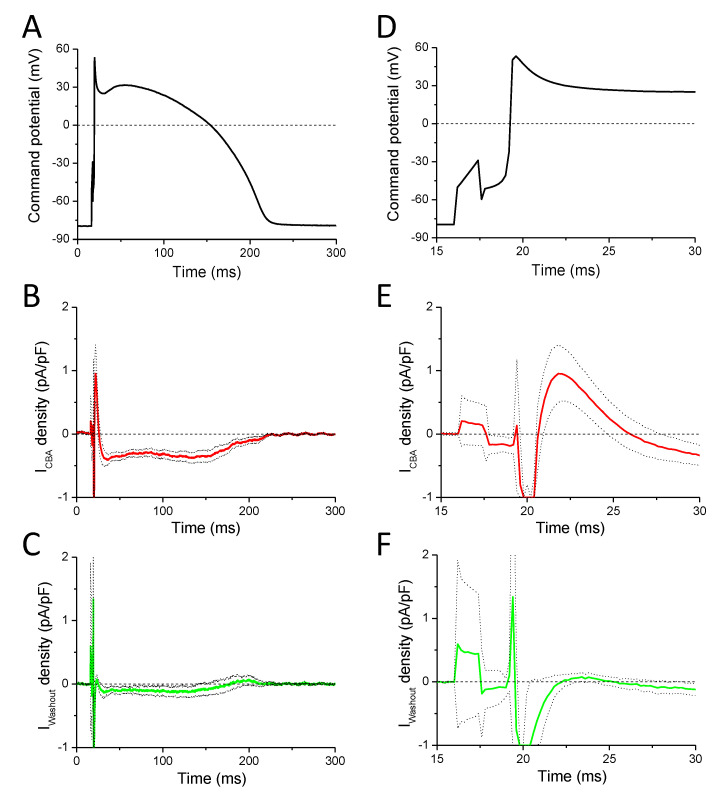
Effects of CBA in action potential voltage-clamp recordings using canonic APs. (**A**) Command potential used for stimulation. (**B**) Average (solid red line) ± SEM (dotted lines) of CBA-sensitive current density obtained in five cells isolated from three animals. (**C**) Average (solid green line) ± SEM (dotted lines) of current densities measured in four out of the initial five cells after washout of CBA. (**D**) The initial part of panel A is shown enlarged. (**E**) The initial part of panel B is shown enlarged. (**F**) The initial part of panel C is shown enlarged.

Based on the results of the previously mentioned experiments, we wanted to be sure that CBA is selective for TRPM4, and the observed effects are not due to actions of the inhibitor evoked on ionic currents other than TRPM4. Therefore, we conducted whole cell patch-clamp recordings with an internal solution containing 10 mM BAPTA to buffer [Ca^2+^]_i_ and therefore prevent the activation of TRPM4 channels. The effect of BAPTA on AP morphology was observed at the beginning of each measurement to confirm its action before starting the application of CBA. CBA-sensitive currents (I_CBA_) were calculated and normalized to cell capacitance as described in the Methods Section 2.5, in five individual cells isolated from three animals. I_CBA_ possessed a short outward component (Figure 5E) during the early repolarization phase of the AP and a much longer inward component during the rest of the AP (Figure 5B). A detailed analysis of the currents is summarized in Table 2, where the results of I_Wout_ current traces (calculated by subtracting the current signals recorded after the washout of 10 µM CBA from those measured in the control condition (in Tyrode solution)) are also indicated. In the case of one cell, the inward peak occurred much later than in others; therefore, it was excluded from averaging. It was detected 141 and 145 ms after the peak of the AP in I_CBA_ and I_Wout_, respectively. The effect of CBA was reversible (Figure 5C,F) as indicated by statistical significance between I_CBA_ and I_Wout_ at certain parameters (Table 2), similarly to most of the actions of CBA on APs recorded with conventional microelectrodes (Figure 2, Figure 3 and Figure 4).

### 2.5. Effects of CBA on Ionic Currents of Repolarization

**Figure 6:**
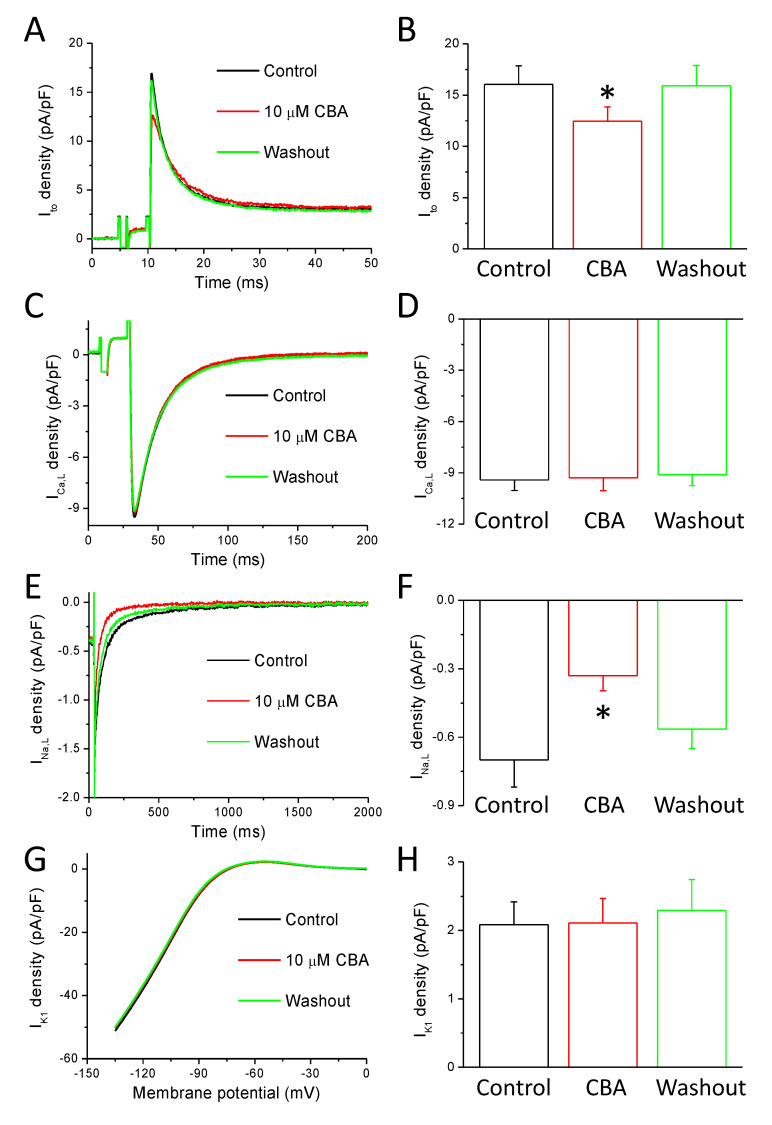
Effects of CBA in ionic currents evoked by conventional voltage-clamp technique. Current traces recorded in control conditions (black), in the presence of CBA (10 µM, red), and after washout of CBA (green). (**A**) Representative I_to_ currents. (**B**) Average ± SEM values of current density values in the indicated conditions of I_to_ obtained in seven examined cells obtained from five animals. (**C**) Representative I_Ca,L_ currents. (**D**) Average ± SEM values of current density values in the indicated conditions of I_Ca,L_ obtained in eight examined cells obtained from four animals. (**E**) Representative TTX-sensitive (I_Na,L_) currents. (**F**) Average ± SEM values of current density values in the indicated conditions of I_Na,L_ obtained in nine examined cells obtained from five animals. (**G**) Representative BaCl_2_-sensitive (I_K1_) currents. (**H**) Average ± SEM values of current density values in the indicated conditions of I_K1_ obtained in seven examined cells obtained from four animals. Asterisks show statistically significant differences compared to the control.

The results of APVC experiments suggested that CBA modulates ion channels other than TRPM4. Based on the CBA-sensitive current profile, the possible targets of CBA-induced inhibition are the transient outward K^+^ current (I_to_) (Figure 6A,B) and the L-type Ca^2+^ current (I_Ca,L_) (Figure 6C,D). Therefore, to selectively study these potential candidates of CBA action, conventional voltage-clamp recordings were conducted. Just like in our APVC experiments presented in Section 2.4, a BAPTA-containing internal solution was used to prevent the activation of TRPM4 channels.

I_to_ was recorded in the presence of 1 µM nisoldipine and 1 µM E4031 in the bath solution in order to block I_Ca,L_ and the rapid component of delayed rectifier K^+^ current (I_Kr_), respectively. I_to_ was activated by a 200-ms-long depolarization to +60 mV from the holding potential of −80 mV at 0.2 Hz stimulation rate and digitized at 10 kHz under software control. Before clamping to the test voltage, a prepulse to −40 mV was applied for 5 ms in order to activate and then inactivate the fast Na^+^ current. I_to_ amplitude was measured as the difference between the peak and the pedestal of the current signal. CBA reduced I_to_ amplitude in the seven studied cells isolated from five animals by 20.4 ± 6.2%, and the current density was 16.0 ± 1.8 pA/pF and 12.5 ± 1.4 pA/pF before and in the presence of CBA, respectively (Figure 6A,B). This inhibitory effect was completely reversible (after the washout of CBA the current amplitude was 15.9 ± 2.0 pA/pF).

During I_Ca,L_ recording the external Tyrode solution contained 1 µM E4031 and 1 µM HMR1556 to block I_Kr_ and the slow component of delayed rectifier K^+^ current (I_Ks_), respectively, as well as 3 mM 4-AP to block I_to_. I_Ca,L_ activation was achieved by 400-ms-long depolarizations to +5 mV arising from the holding potential of −80 mV at 0.2 Hz stimulation rate and digitized at 10 kHz. The test pulse was preceded by a short (20 ms) depolarization to −40 mV to activate then inactivate the fast Na^+^ current. I_Ca,L_ amplitude was measured as the difference between the peak and the pedestal of the current signal. CBA had no effect on the current amplitude, which was −9.4 ± 0.6 pA/pF in control and −9.3 ± 0.8 pA/pF in the presence of CBA measured in eight cells obtained from four animals (Figure 6C,D).

During I_Na,L_ recording, the Tyrode solution contained 1 µM nisoldipine, 100 nM dofetilide, and 100 µM chromanol-293B to eliminate any interference from Ca^2+^ and K^+^ currents. Test pulses were clamped to −20 mV for 2 s from the holding potential of −120 mV at 0.2 Hz stimulation rate and digitized at 5 kHz. The total amount of I_Na,L_ was determined by pharmacological subtraction performed by a final superfusion with 20 µM TTX. The amplitude of I_Na,L_ was evaluated at 50 ms after the beginning of the pulse. For determination of the current integral, the initial 20 ms was excluded from evaluation in order to minimize the contribution of peak sodium current. CBA reduced I_Na,L_ in the nine studied cells isolated from five animals by 47.3 ± 7.0%, as the current amplitude was −0.70 ± 0.12 pA/pF and −0.33 ± 0.07 pA/pF before and in the presence of CBA, respectively (Figure 6E,F). This inhibitory effect was largely reversible (after the washout of CBA, the current density was −0.56 ± 0.08 pA/pF). Similar results were obtained when the integral of I_Na,L_ was determined (total charge values were −160.9 ± 36.1 mC/F, −60.0 ± 16.6 mC/F, and −117.8 ± 21.1 mC/F, in control, in the presence of CBA, and after its washout, respectively).

I_K1_ was measured as a 50 µM BaCl_2_-sensitive current with a 300-ms-long ramp protocol from 0 mV to −135 mV. CBA did not influence I_K1_ in the seven studied cells obtained from four animals, as the current density was 2.09 ± 0.33 pA/pF and 2.11 ± 0.36 pA/pF before and in the presence of CBA measured at −50 mV, respectively (Figure 6G,H). After the washout of CBA, the current density was 2.29 ± 0.44 pA/pF).

## 3. Discussion

### 3.1. TRPM4 Expression in the Canine Heart

TRPM4 is abundantly expressed in many tissues including the heart [5,36,37,38], but this is the first report demonstrating its expression in the canine heart. In many previous studies, only the mRNA expression was described [12,39]. It was similarly present in human and murine hearts [36], and was also detected in rat heart [22,40]. TRPM4 protein is expressed in HEK cells [5,41], in murine sinoatrial nodal tissue [30] and in the plasma membrane of HL-1 cardiomyocytes [42]. In healthy rat ventricle, TRPM4 expression was localized in the plasma membrane [13], and its expression was stronger in atrial than in the ventricular muscle of bovine hearts [9]. In mice, TRPM4 expression was detected at both protein and mRNA levels in atrial and ventricular samples, and also in isolated ventricular cells [14]. In human cardiac tissue, TRPM4 protein expression was described in the conductive system [10], in the atrial muscle [43], and in cardiac samples of 5-month-old Fallot tetralogy patients [40]. TRPM4 mRNA was also present in human left ventricular samples [38].

In the current study, healthy dogs were used to collect cardiac samples. Previously, however, the increase of TRPM4 expression was reported in various pathological conditions such as in rats with spontaneous hypertension [22], in infarcted mouse left ventricle [44], and in NYHA stage 3–4 human heart failure patients [38]. On the contrary, no difference in human atrial TRPM4 expression was found between atrial fibrillation and sinus rhythm patients [43]. Although we could not detect significant differences in TRPM4 expression between the four different chambers, previously, a six-times-higher expression was reported in the right ventricle compared to that of the left in the rat [40]. This might be due to interspecies protein expression differences.

Although cardiomyocytes account for the vast majority of cells in cardiac tissue, excised samples also contain other cell types, including fibroblasts, smooth muscle, and endothelial cells. To minimize the interference of other cell types, the expression of TRPM4 was also determined directly on enzymatically isolated left ventricular cells (far right lane and column of Figure 1). There was no significant difference in TRPM4 expression between isolated cells of the left ventricle and whole left ventricular wall tissue, suggesting that the source of the TRPM4 signal is mostly from cardiomyocytes. Our results are in agreement with a recent study where equal TRPM4 expression was shown in mice atrial and ventricular samples, and in isolated ventricular cardiomyocytes [14].

### 3.2. Effects of CBA on Action Potential Morphology

During AP measurements, we used the conventional sharp microelectrode technique to study the effects of the TRPM4 inhibitor CBA on left ventricular canine cardiomyocytes. This is probably one of the most physiological approaches as it does not compromise the intracellular space of the studied cells. Due to this, neither the buffering of the intracellular Ca^2+^ nor the loss of intrinsic cytosolic Ca^2+^ buffering following cell dialysis is achieved [45]. This is especially important as TRPM4 currents rapidly decrease when measured with whole cell patch-clamp or inside-out patch configurations [6]. In the present study, CBA was used in 10 µM where at least 90% TRPM4 current inhibition was detected without any major action on hERG or L-type calcium channels [35]. This is the first report where the effect of CBA was studied in native cardiac cells—all previous studies were using either TRPM4 knockout animals or other TRPM inhibitors like 9-phenanthrol. Our results indicate that CBA reduced phase-1 slope and increased the action potential amplitude in a reversible manner (Figure 2 and Figure 3). The CBA-induced reduction in maximal rate of depolarization, however, could not be reversed. There was only a tendency for AP shortening but after normalization of the APD_90_ values, to those recorded under control conditions, it resulted in a small (approximately 9%) but significant decrease (Figure 2E). These changes can occur due to either CBA-mediated TRPM4 inhibition or nonspecific actions of CBA evoked on ion channels other than TRPM4, or a combination of these.

In order to hypothesize the possible electrophysiological changes caused by TRPM4 inhibition, we need to imagine how the TRPM4-mediated current might look like under the ventricular AP. For this, the Ca^2+^-dependent activation of TRPM4 [29,30] and its selectivity for monovalent cations [5,12] need to be considered.

In cardiomyocytes, both membrane potential and intracellular Ca^2+^ concentration change dynamically during the cardiac cycle [46]. Moreover, the Ca^2+^ concentrations significantly differ in various cell compartments. The peak Ca^2+^ level may rise to approximately 600 nM in the bulk cytoplasm, to 6 µM in the subsarcolemmal space, or to as high as 10–50 µM in the submembrane cleft (the narrow space between sarcolemmal Ca^2+^ entry and sarcoplasmic reticulum Ca^2+^ release sites) [47,48]. Regarding the Ca^2+^ concentration required for TRPM4 activation, the EC_50_ values are quite variable in the literature, starting from as low as 400 nM [5] and reaching to as high as 1 mM [49], depending on the experimental conditions. In native sinoatrial and ventricular cardiomyocytes, the minimal Ca^2+^ concentration for channel activation was between 0.1–1 µM [22,30,49] and the EC_50_ was 10 µM in rat ventricular cells [22]. Based on these, it is likely that TRPM4 can indeed be activated during the cardiac action potential by the calcium influx. The highest activation is expected shortly after the AP peak and during the early plateau phase [50], because the peak calcium concentration is reached within a few ms after the AP peak in the subsarcolemmal space, and even faster in the submembrane cleft [47]. Based on the fact that TRPM4 is permeable to both Na^+^ and K^+^ (but not to Ca^2+^), the expected reversal potential of its current is around 0 mV [51]. According to these, TRPM4 activation is expected to force the membrane potential towards 0 mV, resulting in a repolarizing current right after the AP peak. Therefore, upon CBA-induced TRPM4 inhibition, the slowing of phase-1 repolarization is anticipated, as seen in our experiments.

The TRPM4-mediated current, on the other hand, could reduce the plateau potential in the early plateau phase, when the membrane potential is higher than 0 mV. Theoretically, the more positive the membrane potential, the larger this reduction will be. Additionally, it might elevate the potential of the late plateau, if the current is still active at this time, but based on the very fast decay of calcium levels in the vicinity of the TRPM4 channels, this is quite unlikely. Therefore, TRPM4 blockade by CBA is expected to somewhat elevate the potential of the early plateau and maybe to very moderately reduce the membrane potential of the late plateau. In our experiments, the plateau potential was not significantly altered by CBA (despite the approximately 2 mV increase of the initially slightly positive Plateau_50_ potential) (Table 1). This can be explained by (i) the nearly 0 value of the Plateau_50_ or (ii) it suggests either the insufficient Ca^2+^ level for TRPM4 activation at this later phase of the AP or (iii) actions of CBA on channels other than TRPM4, being active during that phase.

In theory, a shortening of AP duration is expected from TRPM4 inhibition as observed in rabbit Purkinje fibers in the presence of a different TRPM4 blocker (30 µM 9-phenanthrol) [51] and in murine atrial cells lacking TRPM4 [16,17]. Our results are similar to these, but only a tendency for APD_90_ reduction was observed (approximately 25 ms) and that was only significant after normalization to the initial APD_90_ values of each studied cell, resulting in 9% APD_90_ reduction (Figure 2E). In rabbit ventricular cells, TRPM4 inhibition by 30 µM 9-phenanthrol was, however, without effect on AP amplitude and duration as well as on resting membrane potential and V^+^_max_ [51].

The action of CBA on AP amplitude was statistically significant but very small (3 mV or 2.5% increase) (Figure 2B,E) and might be due to the sum of the non-significant increase and decrease of overshoot potential and resting membrane potential, respectively. From the TRPM4 inhibition during the AP peak, this can be expected, as TRPM4 would tend to move the membrane potential towards 0 mV, therefore antagonizing depolarization, which effect is reduced by CBA, resulting in the increase of overshoot potential and/or AP amplitude. Regarding the maximal rate of depolarization, its CBA-induced reduction might be due to the inhibition of fast Na^+^ current, as V^+^_max_ is a fairly good measure of those channels [52]. However, the relationship between V^+^_max_ and sodium current density is not linear; therefore, a small change in V^+^_max_ can be due to a larger decrease of fast Na^+^ current [53]. The CBA-induced V^+^_max_ reduction was about 15%, not reversible, and not observed in murine ventricular myocardium [16]. A recent study, however, described the smaller value of V^+^_max_ in ventricular myocytes of TRPM4 knockout mice compared to that in wild-type animals [14]. The smaller V^+^_max_ value was due to the reduction of peak Na^+^ current density as it was approximately 30% smaller in TRPM4 knockout mice [14]. It might be that the CBA-induced reduction of V^+^_max_ in our study reflects the reduction peak Na^+^ current, as TRPM4 and Nav1.5 were shown to interact with each other [14]. Our previous results obtained with 9-phenanthrol [34] are in good agreement with the current result regarding the V^+^_max_ and phase-1 slope as both drugs reduced both parameters. In contrast, CBA had no action on either Plateau_50_ or V^−^_max_, unlike the reduction of these by 30 µM 9-phenanthrol [34]. Similarly, the actions of CBA and 9-phenanthrol were different on APD_90_ as a shortening tendency was seen with CBA, but APD reduction was the case in only the minority of the cells with 10 and 30 µM 9-phenanthrol and the prolongation of AP was seen on average [34]. It seems, therefore, that despite the similar TRPM4 inhibition of CBA and 9-phenanthrol, their effects on the canine ventricular AP configuration are markedly different. This is likely due to their different actions on other, non-TRPM4 channels (discussed later).

To summarize, TRPM4 inhibition is expected to result in an increase in APA, slowing of phase-1 repolarization, slight change in plateau potential (elevation in case of positive plateau potentials while depression in case of negative plateau potentials), and shortening of the AP. In our experiments, we detected the small but significant increase of APA, the reduction of phase-1 slope, the irreversible reduction of V^+^_max_, and a tendency for the AP shortening. As these changes can be due to non-TRPM4 actions of CBA, we tested these with ion current recordings and discussed later.

### 3.3. Effects of CBA on Short-Term Variability of Repolarization

TRPM4 channels can mediate transient inward current (like calcium-activated chloride and sodium-calcium exchange currents), leading to DADs in calcium-overloaded cells. These can lead to cardiac arrhythmias by initiating Torsade de Pointes type malignant ventricular tachyarrhytmia [54]. Indeed, TRPM4 inhibition can be antiarrhythmic in human atrial cardiomyocytes and ventricular cells of spontaneously hypertensive rats [12,22]. Similar results were obtained in mouse as 9-phenanthrol abolished hypoxia and re-oxygenation induced early afterdepolarization in a dose-dependent manner [21]. Cardiac specific overexpression of TRPM4 in mice increased the occurrence of ectopic ventricular beats during stress conditions (evoked by heavy exercise and β-adrenergic stimulation), but not in baseline [55], which highlights the possibility that TRPM4 can indeed generate DADs in calcium overloaded cells in vivo. Similarly, TRPM4 could be involved in aldosterone-induced atrial arrhythmias in mice [56]. Moreover, the T160M polymorphism of TRPM4 (together with the G219E of KCNQ1) was found in a family where carriers experienced long QT syndrome [57], which might implicate the role of TRPM4 in the maintenance of normal AP duration. Four TRPM4 mutations were found to be present in cases of sudden cardiac death [58]. A recent review summarizes the role of TRPM4 mutations in inherited cardiac arrhythmia syndromes [59].

We studied the CBA-induced TRPM4 inhibition of short-term variability of repolarization (SV). This marker is a good predictor of cardiac arrhythmias highlighted by a position statement and consensus guidance [60]. As SV reduction might have antiarrhythmic properties, it seems that TRPM4 has a pro-arrhythmic action because its inhibition by CBA caused a reversible reduction of SV (Figure 4A–C). A large, sudden change in consecutive APD values (especially if that happens in a non-uniform manner of the myocardium), can more effectively induce an arrhythmic event [61]. Therefore, in order to detect any unusually short or long APs, consecutive APD values were binned and the overall probability of their appearances was plotted (Figure 4D). The curve was reversibly shifted to the left, supporting again the reduction of chance of cardiac arrhythmia in the presence of CBA. Based on these, it seems that the native TRPM4 current might contribute to cardiac arrhythmia, in agreement with previous studies. Our results, however, suggest a mechanism based on TRPM4 current mediated increase in SV, rather than EAD induction as it was shown previously [21]. It must be mentioned that as CBA might interfere with other ion currents (discussed later), and those could also contribute to the overall effect of CBA on SV as well as on relative SV. Therefore, the reduction of SV might partially be underlain by the CBA-induced reduction of I_to_ and I_Na,L_, as those two currents are likely to increase SV [62] and parallelly, the role of the TRPM4-mediated current in SV increase might be smaller.

### 3.4. Nonspecific Actions of CBA Measured with APVC and Conventional Voltage Clamp

As well as the direct inhibition of TRPM4 channels, CBA-induced changes in AP morphology can also be caused by nonspecific actions of the compound. For instance, the reduction of V^+^_max_ can be due to inhibition of Na^+^ channels, reduction of phase-1 slope, and the increase of APA might be the result of I_to_ inhibition, and the tendency for AP shortening might be due to inhibition of I_Na,L_ or I_Ca,L_ or in theory activation of late K^+^ currents (I_Kr_, I_Ks_, possibly I_K1_). To exclude the possibility of CBA action on ion channels other than TRPM4, we used the whole cell configuration of patch-clamp technique where we applied the fast calcium chelator BAPTA in 10 mM to prevent the activation on TRPM4. This BAPTA concentration is able to fully eliminate not just the intracellular Ca^2+^ transient but also to prevent the systolic rise of subsarcolemmal and probably the submembrane cleft Ca^2+^ levels [45,50]. Indeed, at least in arterial smooth muscle, 10 mM BAPTA was shown to reduce Ca^2+^ levels in microdomains, therefore preventing the activation of TRPM4 [45]. Although not done on direct TRPM4 current recordings, other studies also suggest that BAPTA can block TRPM4 [63,64]. Obviously, it has to be mentioned that the application of BAPTA not only prevents the activation of TRPM4 but also influences all Ca^2+^ sensitive currents including L-type Ca^2+^ current (where its Ca^2+^-dependent inactivation is lost, leading to a higher current) [65], Ca^2+^-activated Cl^−^ current (where its activation is prevented) [50], I_Ks_ (where its otherwise small activation during a normal duration AP is probably further reduced [66]), and I_NCX_ (which will operate in reverse mode, resulting in Ca^2+^ entry leading to an outward current) [65]. These will certainly modify the shape of the AP, but during our measurements, the same canonic APs were always used to stimulate the cells. Actually, the blockade of the Ca^2+^-activated Cl^−^ current and the reduction of the already quite small I_Ks_ were beneficial as CBA were not able to modify those in case of the 10 mM BAPTA-containing AP voltage clamp experiments. During conventional voltage protocols, we isolated the studied currents one-by-one using specific inhibitors and voltage pulses and studied the possible influence of CBA on the given current.

As mentioned before, CBA was only applied after confirming the action of BAPTA on AP contour. The early short outward peak of the CBA sensitive current could be due to CBA induced inhibition of transient outward current flowing during phase-1 (Figure 5E). As the activation of the calcium activated chloride current was prevented with 10 mM BAPTA [50], the inhibited current can only be the transient outward K^+^ current. Indeed, we confirmed this with conventional voltage clamp protocol where all other major currents were either blocked by inhibitors (nisoldipine and E4031) or inactivated by a voltage prepulse (Figure 6A,B). The reversible reduction of I_to_ can account for the outward component of CBA sensitive current of APVC experiments and also fits well with the CBA induced reduction of phase-1 slope and the slight increase of AP amplitude (Figure 2). The long inward current seen in CBA sensitive current might be due to reduction of L-type Ca^2+^ or late sodium current. CBA induced I_NCX_ inhibition is unlikely in our conditions of heavy Ca^2+^ buffering, where that current is mainly outward. CBA had no effect on L-type Ca^2+^ current (Figure 6C,D) in agreement with a previous study [35]. Late sodium current however was reduced by CBA (Figure 6E,F), which can contribute to the small reduction of AP duration seen with AP measurements. As mentioned before a smaller Na^+^ current peak was observed in TRPM4 knockout mice but the late sodium current was not recorded [14]. Nevertheless, it is possible that the late sodium current might also be larger in the presence of TRPM4.

From our results, it is unlikely that CBA interferes with the delayed rectifier K^+^ currents shaping the AP: I_Kr_ and I_Ks_, as the inhibition of these would result in outward currents during the plateau phase of AP measured with APVC. Moreover, if these currents were reduced by CBA the prolongation of the AP could be expected. I_K1_ also plays an important role in terminal repolarization, but we excluded its CBA sensitivity (Figure 6G,H) which is in agreement with no change in the resting membrane potential and in the maximal rate of terminal repolarization seen in AP measurements. In addition, during APVC experiments where TRPM4 could not be activated it would be even more likely to observe these nonspecific inhibitory effects of CBA. Again, there was no diastolic current (indicating a potential I_K1_ blockade) and outward current increasing or reaching peak towards the end of the AP (indicating the potential blockade of I_Kr_ and I_K1_) on the CBA sensitive current traces. As neither AP changes nor the CBA sensitive current suggested potential inhibition of I_K1_, I_Kr_ and I_Ks_ we can rule out the CBA inhibition of these three K^+^ currents even in the absence of direct current measurements in case of I_Kr_ and I_Ks_. The lack of CBA action on I_Kr_ could be expected as cardiac hERG channels (the pore forming subunit of I_Kr_) were not influenced by CBA as less than 5% reduction was detected by 10 µM CBA judged by reduction in specific antagonist (dofetilide) binding [35].

Comparing our previous study done with 9-phenanthrol, we can conclude that CBA is more specific than the widely applied 9-phenanthrol, as CBA influenced only I_to_ and I_Na,L_ whereas 9-phenanthrol reduced all major K^+^ currents (I_to_, I_Kr_ and I_K1_) in concentrations used to inhibit TRPM4 [34]. Although neither 9-phenanthrol nor CBA reduced L-type Ca^2+^ current, 9-phenanthrol activated an extra current in the voltage range between +10 and +40 mV [34] further adding to its low specificity.

One more thing about CBA requires discussion: its chemical chaperone activity. It was observed that overnight application of high (50 µM) dose of CBA (but not its inactive congener) partially restored the functional expression and the Ca^2+^ sensitive outward current in cell transfected with the loss of expression mutant A432T TRPM4 channel. Similarly, increased expression was observed with wild-type TRPM4 channels after long and high dose CBA pretreatment (50 µM, overnight) [35]. These actions are unlikely to occur in our experiments, as CBA was applied in lower concentration (10 µM) and for a much shorter time (5 min).

### 3.5. Summary and Potential Relevance

The major findings of the present study indicate the expression of TRPM4 protein in all four chambers of the canine heart as well as in isolated left ventricular canine cardiomyocytes. CBA, a fairly new compound, seems to be more specific than 9-phenanthrol to inhibit TRPM4 and therefore to study its role in cardiac physiology. As CBA influences I_to_ and I_Na,L_, extra care must be taken when using it in such experimental conditions where these ion currents are active, such as in native cardiomyocytes.

## 4. Methods

### 4.1. Isolation of Canine Ventricular Myocytes

Cell isolation was carried out with segment perfusion technique by enzymatic digestion as described previously [50]. Intramuscular application of 10 mg/kg ketamine hydrochloride (Calypsol, Richter Gedeon, Budapest, Hungary) and 1 mg/kg xylazine hydrochloride (Sedaxylan, Eurovet Animal Health BV, Bladel, The Netherlands) was used to achieve complete narcosis in adult mongrel dogs of either sex according to protocols approved by the local ethical committee (license No.: 9/2015/DEMÁB) in line with the ethical standards laid down in the Declaration of Helsinki in 1964 and its later amendments as well as with the Guide to the Care and Use of Experimental Animals (Vol. 1, 2nd ed., 1993, and Vol. 2, 1984, Canadian Council on Animal Care). Chemicals and reagents were purchased from Sigma-Aldrich Co. (St. Louis, MO, USA) if not specified otherwise. Hearts were quickly removed in left lateral thoracotomy and washed in cold Tyrode solution containing (in mmol/L): NaCl 144, KCl 5.6, CaCl_2_ 2.5, MgCl_2_ 1.2, 4-(2-Hydroxyethyl)piperazine-1-ethanesulfonic acid (HEPES) 5, glucose 10 (pH = 7.4; adjusted with NaOH). Left anterior descending coronary artery was cannulated and perfused with a nominally Ca^2+^-free JMM solution (Minimum Essential Medium Eagle, Joklik Modification, product no. M0518) gassed with a mixture of 95% O_2_ and 5% CO_2_ and supplemented with 2.5 g/L taurine, 200 mg/L NaH_2_PO_4_, 1.4 g/L NaHCO_3_, 175 mg/L pyruvic acid, 13.5 mg/L allopurinol, and 750 mg/L D-ribose, pH 6.8, and heated to 37 °C. Then, atria were cut off and a wedge-shaped section of the left ventricular wall supplied by the left anterior descending coronary artery was perfused. Following further 5 min of perfusion to completely remove blood from the tissue, 0.9 g/L collagenase (type II, 245 U/mg; Worthington Biochemical Co., Lakewood, NJ, USA), 2 g/L bovine serum albumin (Fraction V.), and 50 µM CaCl_2_ were added to the JMM solution. During the 30–40 minutes-long enzymatic digestion, the solutions were kept at 37 °C and gassed with a mixture of 95% O_2_ and 5% CO_2_. Cells were sedimented and filtered four times to remove big chunks. During this procedure, the Ca^2+^ concentration of the JMM solution was gradually restored to the final 1.8 mmol/L. After this, cells were stored in MEM solution (Minimum Essential Medium Eagle, product no. M0643) supplemented with the followings: 2.5 g/L taurine, 200 mg/L NaH_2_PO_4_, 2.2 g/L NaHCO_3_, 175 mg/L pyruvic acid, 13.5 mg/L allopurinol, 750 mg/L D-ribose (pH = 7.3, equilibrated with a mixture of 95% O_2_ and 5% CO_2_) at 15 °C until further use within 36 h after isolation. The percentage of living cells (having clear cytoplasm, sharp edges and clear striations) were usually 30–60% and only these were used for experiments.

### 4.2. Electrophysiology

Cells were placed in a plexiglass chamber with a volume of 1 mL and continuously superfused with bicarbonate buffer containing Tyrode solution containing (in mmol/L): NaCl 121; KCl 4; MgCl_2_ 1; CaCl_2_ 1.3; HEPES 10; glucose 10; NaHCO_3_ 25; (pH=7.3; adjusted with NaOH) supplied by a gravity driven system at a speed of 2 mL/min. During experiments the bath temperature was set to 37 °C by a temperature controller (Cell MicroControls, Norfolk, VA, USA). Cells were visualized by inverted microscopes placed in a Faraday cage on an anti-vibration table (Newport, Rochester, NY, USA). Electrical signals were recorded with intracellular amplifiers (MultiClamp 700A or 700B, Molecular Devices, Sunnyvale, CA, USA) after analogue-digital conversion (Digidata 1440A or 1332, Molecular Devices, Sunnyvale, CA, USA) and recorded with pClamp 10 software (Molecular Devices, Sunnyvale, CA, USA). Cells were perfused with 10 µM CBA (Tocris Bioscience, product no. 6724) for 5 min followed by a 5-min-long period of washout. CBA was dissolved in DMSO so the final DMSO concentration was 0.1%, which did not affect any of the physiological parameters studied.

### 4.3. Recording of Action Potentials

Action potentials (APs) were measured with 3 mol/L KCl containing borosilicate microelectrodes having a tip resistance of 20–50 MΩ. 1 s cycle length steady-state pacing was achieved supra-threshold current pulses (1–2 ms long, 120–130% of threshold) produced by an electronic stimulator (DS-R3; Főnixcomp Ltd, Debrecen, Hungary). APs were digitized at 50 kHz and upon the off-line analysis of APs the following parameters were determined in ten consecutive APs then averaged: APD_50_, APD_75_, and APD_90_ values (duration of the AP from the peak to 50, 75, and 90% of repolarization, respectively), maximal rate of phase 0, 1, and 3 (V^+^_max_, phase-1 slope, and V^−^_max_, respectively), resting membrane potential (RMP), overshoot potential (OSP), APA (action potential amplitude, determined as the difference between OSP and RMP), and membrane potential at the half duration of APD_90_ (Plateau_50_).

### 4.4. Analysis of Variability of AP Repolarization

A series of 50 consecutive action potentials was recorded as described earlier and analyzed off-line to estimate short-term variability of repolarization (SV) using the following formula:

$$\mathrm{SV}=\frac{\sum_{i=1}^{n}\left(\left|APD_{i+1}-APD_{i}\right|\right)}{n\surd 2}$$

where SV is short-term variability, *APD_i_* and *APD_i_*_+1_ indicate the APD_90_ values of the *i*th and (*i* + 1)th APs, respectively, and *n* denotes the number of consecutive beats analyzed [62,67]. Poincaré diagrams made out of 50 consecutive APD_90_ values were used to visualize CBA-induced changes in SV. As the value of SV strongly depends on the value of APD_90_ [62], SV can be best judged as a function of APD. To further analyze repolarization variability, the differences between consecutive APD_90_ values were grouped in ranges (below 20 ms in 1 ms ranges and above 20 ms differences and 5 ms ranges) and the overall probability of their appearance was calculated in each cell [68]. Then the average of these data was plotted (see Figure 4D) to illustrate the changes in beat-to-beat variability of APD.

### 4.5. Voltage-Clamp Studies

Membrane currents were recorded using the patch-clamp technique [69] in whole-cell configuration. The cells were superfused with bicarbonate buffer containing Tyrode solution (see above for composition) at 37 °C. Borosilicate glass micropipettes had tip resistance of 2–3 MΩ after filling with pipette solution containing (in mmol/L): K-aspartate 100; KCl 45; BAPTA 10; HEPES 5; K_2_ATP 3; MgCl_2_ 1; KOH 10. The pH of the pipette solution was adjusted to 7.3 using KOH and its osmolality was 287–290 mmol/kg measured with a vapor pressure osmometer (Vapro 5520, Wescor Inc., Logan, UT, USA). After establishing high (1–10 GΩ) resistance seal by gentle suction, the cell membrane beneath the tip of the electrode was disrupted by further suction and/or by applying 1.5 V electrical pulses for 1 ms. The series resistance was typically 4–6 MΩ before compensation (usually 50–80%). Experiments were discarded when the series resistance was high or substantially increased during the measurement. Ion currents were normalized to cell capacitance using short hyperpolarizing pulses to −10 mV for 45 ms from 20-ms-long depolarization to 0 mV from the holding voltage of −80 mV applied at 10 Hz. The average value of cell capacitance was 144.1 ± 3.8 pF in the average of the 41 myocytes studied. The voltage protocol applied in the experiments and any difference in solutions is described where pertinent in the Results section.

Action potential voltage-clamp experiments were conducted according to the method described previously [50,70] and signals were digitized at 50 kHz. In the experiments, a previously recorded typical AP (recorded with 700-ms-long cycle length pacing on a midmyocardial cell, termed as “canonic” AP) was applied to the voltage-clamped cells as a command signal. I_CBA_ was obtained by pharmacological subtraction calculated by deducting the current signals recorded in the presence of 10 µM CBA from those measured in control condition (in Tyrode solution) [50,70].

### 4.6. Protein Sample Preparation and Western Blot Analysis

For Western blot experiments total cell lysates were prepared from canine left ventricular cardiomyocytes (generated as described earlier (Section 2.1)) and from tissue samples (excised from the wall of the four chambers of the heart) by mechanical force methods. Briefly, cell lysates from the tissue samples were prepared with destruction of the cells by stainless steel balls, while cardiomyocytes were subjected to sonication. Protein concentration was determined by BCA protein assay (Thermo Scientific, Rockford, IL, USA) then samples were subjected to SDS-PAGE (10% gels loaded with 20 μg protein per lane) and transferred to nitrocellulose membranes (Bio-Rad, Hercules, CA, USA). Membranes were blocked with 5% dry milk–PBS solution and were probed with anti-TRPM4 primary antibodies (OriGene Technologies, Rockville, MD, USA, TA500381, clone OTI10H5, mouse-IgG, polyclonal, 1:1500) followed by HRP-conjugated secondary antibody labeling in 1:1000 dilution. The primary–secondary antibody complexes were detected using an enhanced chemiluminescence Western blotting Pico or Femto kit (Thermo Scientific, Rockford, IL, USA) in a Fujifilm Labs-3000 dark box. Membranes were then stripped and subjected to α-actinin-specific labeling (1:1000; Santa Cruz BioTechnology, Dallas, USA). To quantify expression levels, background-corrected densitometry was performed using ImageJ (NIH, Bethesda, MD, USA). The optical densities of the TRPM4-specific bands were normalized to those of the α-actinin-specific ones of the samples.

### 4.7. Statistics

All values are presented as arithmetic means ± Standard Error of the Mean (SEM). Given the biological variability among cells, each cell was treated as independent in the statistical tests, although more cells could be obtained from the same animal. In the manuscript, *n*/*n* values are used to indicate the number of the studied cells/total number of animals from where isolation of these cells was made. Statistical significance of differences was evaluated using one-way ANOVA followed by Student’s *t*-test. Differences were considered significant when *p* was less than 0.05 and indicated with asterisks on graphs.

## Figures and Tables

**Table 1:** Unaltered AP parameters by CBA recorded from eight individual cells obtained from six animals.

	RMP (mV)	OSP (mV)	Plateau_50_ (mV)	V^−^_max_ (V/s)
In control condition	−83.6 ± 0.9	31.0 ± 2.1	1.3 ± 3.0	−1.5 ± 0.1
In the presence of 10 µM CBA	−85.2 ± 1.4	32.4 ± 1.9	3.5 ± 4.6	−1.6 ± 0.1
After washout of CBA	−84.8 ± 1.5	29.7 ± 1.7	3.4 ± 4.1	−1.5 ± 0.1

**Table 2:** Parameters of CBA- and washout-sensitive currents in APVC experiments. Significant differences between the values of I_CBA_ and I_Wout_ of the given parameters are indicated with asterisks in front of the name of the parameter.

	I_CBA_	I_Wout_
* Early outward peak current density (pA/pF)	1.38 ± 0.32 (*n* = 5)	0.16 ± 0.06 (*n* = 4)
Time of early outward peak measured from the peak of the AP (ms)	2.33 ± 0.48 (*n* = 5)	3.49 ± 0.47 (*n* = 4)
Charge carried by outward component (fC/pF)	3.94 ± 1.58 (*n* = 5)	0.54 ± 0.22 (*n* = 4)
* Inward peak current density (pA/pF)	−0.53 ± 0.06 (*n* = 5)	−0.25 ± 0.07 (*n* = 4)
Time of inward peak after measured from the peak of the AP (ms)	11.73 ± 0.67 (*n* = 4)	9.41 ± 3.34 (*n* = 3)
* Charge carried by inward component (fC/pF)	−55.33 ± 11.42 (*n* = 5)	−19.68 ± 9.03 (*n* = 4)
* Inward current density at measured at Plateau_50_ of the AP (pA/pF)	−0.35 ± 0.07 (*n* = 5)	−0.13 ± 0.09 (*n* = 4)

## Data Availability

Data sharing not applicable.

## Abbreviations

4-AP	4-aminopyridine
AP	action potential
APA	action potential amplitude
APD	action potential duration
APD_90_	action potential duration at 90% of repolarization
APVC	action potential voltage-clamp
CBA	4-chloro-2-(2-chlorophenoxy)acetamido) benzoic acid
[Ca^2+^]_i_	intracellular Ca^2+^ concentration
DAD	delayed afterdepolarization
EAD	early afterdepolarization
EC_50_	half effective activator concentration
I_Ca,L_	L-type Ca^2+^ current
I_CBA_	CBA-sensitive current
I_Cl(Ca)_	Ca^2+^-activated Cl^−^ current
I_K1_	inward rectifier K^+^ current
I_Kr_	rapid component of delayed rectifier K^+^ current
I_Ks_	slow component of delayed rectifier K^+^ current
I_Na,L_	late Na^+^ current
I_NCX_	Na^+^/Ca^2+^ exchange current
I_ti_	transient inward current
I_to_	transient outward K^+^ current
OSP	overshoot potential
Phase-1 slope	maximal rate of early repolarization
PIP2	phosphatidylinositol 4,5-bisphosphate
Plateau_50_	membrane potential at the half duration of APD_90_
RMP	resting membrane potential
RSV	relative short-term variability of repolarization
SV	short-term variability of repolarization
TRP	transient receptor potential
TRPM4	transient receptor potential melastatin 4
V^+^_max_	maximal rate of depolarization
V^−^_max_	maximal rate of terminal repolarization
